# An *in vitro *co-culture model of esophageal cells identifies ascorbic acid as a modulator of cell competition

**DOI:** 10.1186/1471-2407-11-461

**Published:** 2011-10-25

**Authors:** Lauren MF Merlo, Rachelle E Kosoff, Kristin L Gardiner, Carlo C Maley

**Affiliations:** 1 The Wistar Institute, 3601 Spruce St., Philadelphia, PA 19104, USA; 2 Cell and Molecular Biology Program, University of Pennsylvania, 451 Curie Blvd, Philadelphia, PA 19104, USA; 3 Helen Diller Family Comprehensive Cancer Center, Department of Surgery, University of California, San Francisco, 2340 Sutter St., San Francisco, CA 94115, USA

## Abstract

**Background:**

The evolutionary dynamics between interacting heterogeneous cell types are fundamental properties of neoplastic progression but can be difficult to measure and quantify. Cancers are heterogeneous mixtures of mutant clones but the direct effect of interactions between these clones is rarely documented. The implicit goal of most preventive interventions is to bias competition in favor of normal cells over neoplastic cells. However, this is rarely explicitly tested. Here we have developed a cell culture competition model to allow for direct observation of the effect of chemopreventive or therapeutic agents on two interacting cell types. We have examined competition between normal and Barrett's esophagus cell lines, in the hopes of identifying a system that could screen for potential chemopreventive agents.

**Methods:**

One fluorescently-labeled normal squamous esophageal cell line (EPC2-hTERT) was grown in competition with one of four Barrett's esophagus cell lines (CP-A, CP-B, CP-C, CP-D) under varying conditions and the outcome of competition measured over 14 days by flow cytometry.

**Results:**

We demonstrate that ascorbic acid (vitamin C) can help squamous cells outcompete Barrett's cells in this system. We are also able to show that ascorbic acid's boost to the relative fitness of squamous cells was increased in most cases by mimicking the pH conditions of gastrointestinal reflux in the lower esophagus.

**Conclusions:**

This model is able to integrate differential fitness effects on various cell types, allowing us to simultaneously capture effects on interacting cell types without having to perform separate experiments. This model system may be used to screen for new classes of cancer prevention agents designed to modulate the competition between normal and neoplastic cells.

## Background

Cancer progression is an evolutionary process by which heterogeneous populations of neoplastic clones compete with each other and normal cells for space and resources [1]. All interventions, whether preventive or therapeutic, are attempts to perturb this process of clonal evolution. Ultimately, if a treatment kills or disrupts neoplastic cells, some cell type must grow back in their place. Our interventions are implicit attempts to bias this competition in favor of normal cells. Successful prevention and therapeutic interventions can modulate the dynamics of competition in one of two ways, either

1) neoplastic cells may be negatively affected by a therapy or intervention, thus reducing the competitive advantage of these cells relative to normal cells. Most traditional interventions employ this strategy of reducing the fitness of neoplastic cells by killing or preventing proliferation. Alternatively,

2) the "normal" cells may gain a competitive advantage from a mitogen or survival factor added to the neoplastic environment that differentially affects cell fitness, allowing the normal cells to outcompete the neoplastic cells, a strategy we refer to as "benign cell boosters" [2]. Computational models suggest this may be an effective strategy to harness clonal competition to prevent cancer [2].

Clear documented examples of clonal expansion [3-6] demonstrate that there is interaction and competition between heterogeneous clones within a neoplasm and those clones may displace normal cells in a tissue. Although competition between heterogeneous cell types is a fundamental property of progression and therapeutic intervention [7-9], the mechanism of competition is incompletely understood and only a few studies [10-12] have attempted to directly quantify the dynamics of competition between normal and neoplastic cells [13]. Here, we define competition as interaction between two cell types such that the cell types exhibit behavior or dynamics when together that is not present when each cell type is grown alone. This is based on an ecological definition of competition, where the fitness of one population negatively affects the fitness of another, and can be the result of both changes in proliferative or death processes. Early work by Heppner and Miller demonstrated that subpopulations of mouse mammary tumor cells could affect each other's growth when reinjected into mice [14]. More recent studies of cell competition in cancer have found that cells containing a mutant tumor suppressor *lgl *or a mutant *lgl*-binding protein, *mahj*, can be competitively eliminated [15]. Indirect measures from human neoplasms suggest that oncogenic mutations may only increase clone relative fitness by 0.5% in clonal competition [16]. Transformed cells have also been found to exhibit different behavior when surrounded by normal cells compared to other transformed cells [17-19]. In Drosophila, cells containing extra copies of the *myc *proto-oncogene can outcompete wild-type cells [20]. While there is certainly extensive interest in competition in cancer [13,21,22], cell competition plays an important role in other cellular systems, such as the developmental programme of D*rosophila melanogaster *[23-25]. In cancer studies, most standard *in vitro *systems do not include normal cells or multiple neoplastic cell types and thus fail to model the process of competition that is the true target of our interventions. Here, we have developed a cell culture model system in which competition dynamics can be directly measured.

Barrett's esophagus (BE) provides an ideal model in which to test the evolutionary dynamics of competition. In an environment of chronic gastroesophageal reflux, in some patients, BE cells (specialized intestinal metaplasia) replace normal squamous tissue in the distal esophagus [26]. Acid suppression alone is not sufficient to allow the squamous cells to outcompete Barrett's cells and cause regression, though the combination of wounding, via biopsies or ablation, along with acid suppression, can lead to regrowth of squamous tissue [26-28]. BE is of clinical importance because it is the only known precursor of esophageal adenocarcinoma (EA) and is associated with a relative risk of esophageal adenocarcinoma of 30-125 compared to the general US population of similar age [29]. However, the natural history of BE is typically nonprogressive, with the risk of progression to EA approximately 0.6-0.7%/year [30,31]. Thus, there may be an extensive time period in which to intervene in the process of progression to prevent EA and a unique opportunity for chemoprevention given that patients likely have BE for many years before EA develops. BE lacks a physiologically realistic animal model system for investigating potential chemopreventive agents, the best known being a surgical anastomosis model in the rat [32-36]. Therefore, there is a need for a simple model system for screening compounds that may affect the establishment and progression of BE.

We have developed a sensitive *in vitro *model of competition between Barrett's esophagus and squamous esophageal cells that may be used to identify a new class of interventions, explicitly designed to modulate competition in favor of normal cells. Co-cultures of an esophageal squamous cell line with each of four BE cell lines were evaluated over 14 days for changes in the proportion of cells of each population under varying concentrations of the antioxidant vitamin C, as well as vitamin E and epidermal growth factor [37,38]. In addition to exposure under normal cell culture growth conditions, we also examined the effect of daily acid pulses on vitamin C competitions to better replicate the reflux conditions of the lower esophagus in patients with BE. Results demonstrate that vitamin C reduces growth of BE cells relative to normal squamous cells, giving a competitive advantage to normal squamous cells at physiologically relevant concentrations of vitamin C. Under acidic conditions, the advantage conferred to normal cells by vitamin C is generally greater. This is consistent with recent epidemiological data suggesting that vitamin C may help prevent progression from Barrett's esophagus to esophageal adenocarcinoma [39,40].

## Methods

### Cell Lines

Four hTERT transformed Barrett's esophagus (BE) cell lines (gift of P. Rabinovitch, University of Washington, Seattle WA) were used in competition experiments. These cell lines, CP-A, CP-B, CP-C, and CP-D, were established from BE cells isolated from 4 different patients. The CP-A cell line (9p LOH, TP53^wt^, 5q LOH) was established from an area of nondysplastic metaplasia and the CP-B (9p LOH, 17p LOH, TP53^mut^), CP-C (9p LOH, 17p LOH, TP53^mut^), and CP-D (9p LOH, 17p LOH, TP53^mut^) cell lines were established from areas of high-grade dysplasia. All 4 BE lines are aneuploid [41,42]. Normal esophageal squamous cells that had been hTERT transformed (EPC2-hTERT) were also used (gift from A. Rustgi, University of Pennsylvania, Philadelphia PA). All BE cell lines were adapted to serum-free conditions in keratinocyte serum-free (KSF) medium supplemented with bovine pituitary extract and epidermal growth factor (EGF) (Invitrogen Corp. Carlsbad, CA) by serial passaging of cell lines into successively lower concentrations of serum until the cell lines survived without serum present. BE cell line identities were verified with the Identifiler PCR Amplification kit (Applied Biosystems).

### Cell Labeling

Esophageal squamous cells (EPC2-hTERT) were transduced with an eGFP-expressing lentivirus (gift of M. Herlyn, Wistar Institute, Philadelphia, PA). Transduced cells were sorted twice by flow cytometry to purify eGFP expressing cells. The stability of the fluorescent tag was monitored throughout each experiment, and it was maintained at 100% of the original fluorescence for 14 days, the duration of competition experiments. Labeled EPC2 cell lines were tested for evolutionary neutrality of the fluorescent label by competition between labeled and unlabeled populations of the EPC2 cells to confirm that the labeled cells do not generate a differential response to the mitogen of interest. We verified that there is no interaction between ascorbic acid and the eGFP fluorescent label (Additional File 1, Figure S1).

### Co-culture of squamous and BE cells without acid

Competitions consisted of one unlabeled (non-fluorescent) BE cell line and one eGFP-labeled squamous cell line. These were seeded with a total of 2 × 10^5 ^cells (50% from each cell line) into a 60 mm plate (Greiner, USA Scientific). Initial seeding ratios were measured by flow cytometry. Cells were cultured in KSF medium (Invitrogen) with 50 U/mL penicillin and 50 ug/mL streptomycin supplemented with the agent of interest for 14 days, with media changes every 2-3 days. Vitamin C (ascorbic acid, Fisher Scientific) stocks were made every 5 days at 1000X the final concentration in PBS and filter sterilized (0.22 μm filter). Competitions were performed at 0, 50, 150, and 500 μM concentrations of ascorbic acid to approximate physiologically relevant conditions based on plasma and mucosal concentrations of this compound [43,44]. The effect of two other compounds, α-tocopherol (Sigma) and EGF (Gibco), was measured using an identical protocol (For methods, see Additional File 1, Figures S2, S3). Competitions were passaged by complete trypsinization every 3-4 days. 0.25% Trypsin (Gibco) was inhibited with addition of 250 mg/L soybean trypsin inhibitor (Gibco) in PBS. Trypsinized plates were examined by light microscopy to verify that no cells still adhered to the plate. For each experiment, there were 4 sampling points, starting 3-4 days after initial seeding. Competitions were reseeded with 20% of the cells after trypsinization. Flow cytometric analysis (Beckman Coulter EPICS XL flow cytometer) was performed on days 3, 7, 10 and 14 after seeding to discriminate between the fluorescent normal squamous cells and the non-fluorescent BE cells.

### Acid Pulsing of co-cultured squamous and BE cells

Co-culture methods are as described above with the exception that for acid pulsed cells, competitions were pulsed for 6 minutes daily with pH 3.5 PBS at 37°C, rinsed twice with neutral pH PBS and fresh media was replaced in each plate. These competitions were reseeded with 15% of the cells after trypsinization and flow cytometric analysis (Beckman Coulter EPICS XL flow cytometer) performed on days 3, 7, 10 and 14 after seeding.

### Growth of Cell Lines in Monoculture

For each of the 4 BE cell lines (CP-A, CP-B, CP-C, CP-D) and 1 esophageal squamous cell line (EPC2), 10,000 cells were seeded into a 6-well plate containing KSF media (Invitrogen) with or without 100 μM ascorbic acid. After 6 days of growth, cells were trypsinized and counted (Nucleocounter, New Brunswick Scientific).

### Immunofluorescence of Proliferation and Apoptosis

#### Fixation of cells

Cells from competition experiments were fixed with 2% PFA in PBS for 20 minutes at room temperature, centrifuged at 485 × g for 5 minutes, and washed twice with PBS. PFA was made from a 16% stock solution (Electron Microscopy Sciences, Hatfield, PA) once per month and stored at 4°C in the dark. Fixed cells were stored in the dark at 4°C in PBS until analysis.

#### Phospho-HistoneH3

PHH3 was probed as a marker of proliferation. Fixed cells were blocked with 5% goat serum for 30 minutes at room temperature. After 2 rinses with a wash buffer of 0.1% saponin (Acros) in PBS, the primary antibody (Sigma H0412) was added to each sample at a dilution of 1:500 in the wash buffer. After 1 hour at room temperature, 3 washes were performed. The secondary antibody (goat anti-rabbit Alexa Fluor 488) was added at 1:100 dilution in wash buffer. This was followed by 3 more washes. Samples were analyzed by flow cytometry (Beckman Coulter EPICS XL flow cytometer). Post-acquisition analysis was performed with Flowjo (Version 8.7.1).

#### Active Caspase-3

The cleaved, active form of Caspase-3 (Sigma C8487) was probed as a marker for apoptosis at a single time point at the end of the competition period. The protocol followed was the same as for PHH3, with a 1:500 dilution of the primary antibody and the same secondary antibody used at 1:100dilution. Samples were run on the flow cytometer (Beckman Coulter EPICS XL flow cytometer) and post-acquisition analysis was performed with Flowjo (Version 8.7.1).

#### Ethidium Homodimer-1

To measure total cell death, time lapse imaging of cells in monoculture and competition was performed using a nonspecific marker for cell death, ethidium homodimer-1 (Invitrogen). This assay has the advantage of measuring all forms of cell death, not just apoptosis. Competitions were established in 6-well plates using the methodology described above and 2.5 μM ethidium homodimer-1 added to the culture 24 hours following cell seeding. Immediately following the addition of the ethidium homodimer, plates were imaged using a Nikon TE300 inverted microscope equipped with a motorized XY stage and Environmental chamber (Temperature and CO_2 _control surrounding entire microscope). Image Pro 6.2 was used for image acquisition and also controls the motorized stage, filter wheels, shutters, and lamp settings. Using the automated stage macro, twelve areas selected, 2 fields per well, locations in any X, Y, or Z direction saved. Two images from the same position in each well were taken every 15 minutes for a total of 48 hours with a Q-imaging retiga EX digital camera. Percent death statistics were calculated by manually tracing cell fates in each time laps experiment for 240-455 cells per condition.

### Statistical Analysis

Statistical significance was evaluated using the R statistics software by a series of t-tests comparing the difference in proportion of BE cells between day 0 and day 14 at 0 μM and 500 μM Vitamin C. Using a Bonferroni correction for multiple testing, comparisons with a p-value less than 0.00625 were considered significant.

## Results

### Competitions under varying levels of Vitamin C

**Figure 1:**
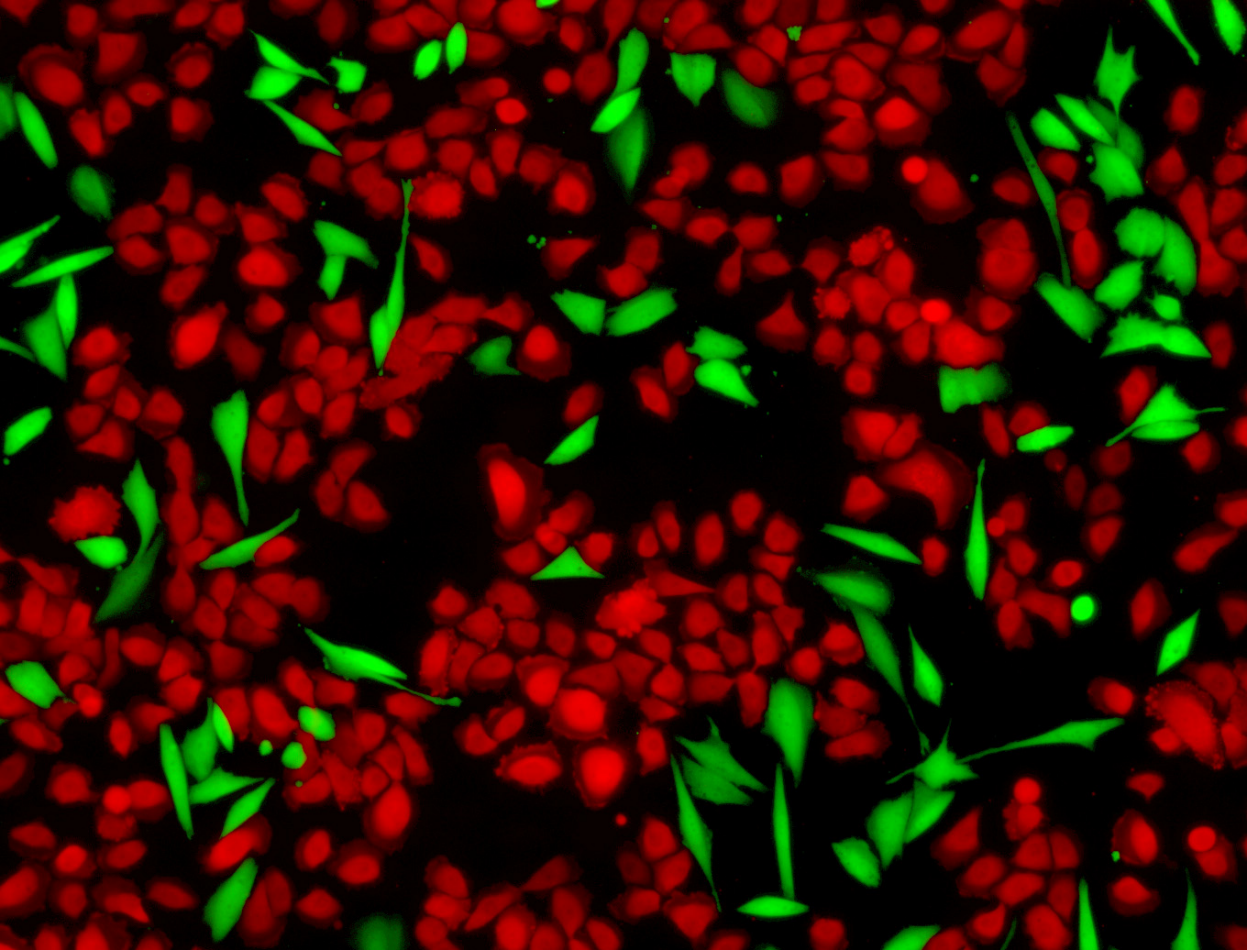
**Cell culture competition**. Image of competition between Barrett's esophagus cell line CP-D (green) and normal squamous cell line EPC2 (red). Cells are grown until they begin to approach confluency and are then passaged to maintain logarithmic growth. Both cell lines are labeled here for imaging purposes, however, in standard competitions BE cell lines are unlabeled. Note that the green cells do not necessarily grow in contiguous patches, and so there must either be cell migration or a process of detachment and reattachment as those clones expand. The two cell types can interact both by physical contact and via secreted factors.

**Figure 2:**
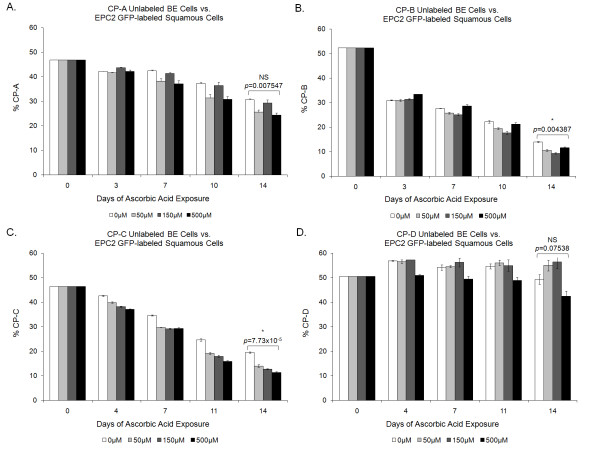
**Competition results**. All four BE cell lines show a trend towards reduced proliferation with 500 uM Vitamin C compared to 0 uM controls, but the effect is only statistically significant for CP-B and CP-C cell lines when adjusted for multiple testing using a Bonferroni correction (p < 0.05/8 = 0.00625 required for significance). Mean (n = 3) ± standard error of the mean is shown.

**Figure 3:**
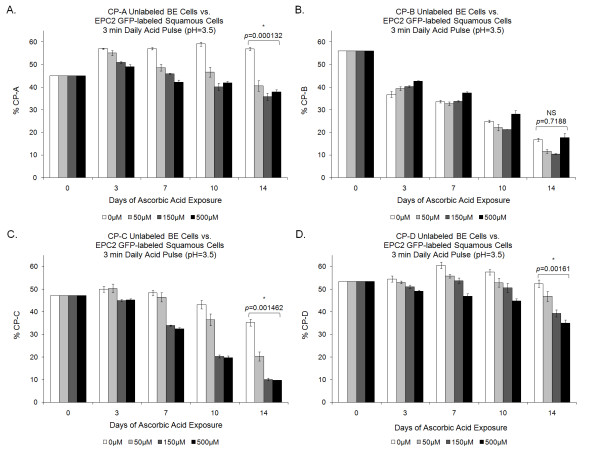
**Acid-pulsed competition results**. Competition experiments were subjected to daily acid pulsing to model gastric reflux in Barrett's esophagus. Under acid pulsing, CP-A, CP-C, and CP-D BE cell lines show a statistically significant reduction relative to normal squamous cells with 500 μM Vitamin C compared to 0 μM controls when adjusted for multiple testing using a Bonferroni correction (p < 0.05/8 = 0.00625 required for significance). CP-B cells show a reduction at lower concentrations of vitamin C but not at 500 μM. Mean (n = 3) ± standard error of the mean is shown.

Four unlabeled BE cell lines (CP-A, CP-B, CP-C, and CP-D) were tested in co-culture competition (Figure 1) against a stably-transduced eGFP-labeled normal esophageal squamous cell line (EPC2). The effect of vitamin C exposure varied between cell lines. There is a statistically significant reduction in BE cells at 500 μM ascorbic acid relative to the 0 μm treatment for CP-B and CP-C cell lines (Figure 2B, C), demonstrating that the presence of Vitamin C gives the EPC2 squamous cells a relative competitive advantage. This trend is also apparent in the CP-A cell line (Figure 2A), although this is not statistically significant under the conservative Bonferroni multiple testing correction. While we find that squamous cells more rapidly outcompete BE cells in the presence of vitamin C, this effect is often not dose-dependent. The CP-B cell line shows a particularly noteworthy saturation effect in both the no-acid and acid-pulsed competitions (Figure 2B, 3B), with lower concentrations of ascorbic acid providing a greater competitive advantage to the squamous cells.

### Acid-pulsed competitions under varying levels of Vitamin C

To better simulate the phenomenon of acid reflux associated with BE (a potential cause of the condition), competitions were exposed to pH 3.5 PBS for 6 minutes daily. With daily acid pulses, the difference between the 0 and 500 μM ascorbic acid treatments is generally magnified, with CP-A, CP-C, and CP-D cell lines all showing a decrease in BE cell lines with Vitamin C present (Figure 3). This effect occurred in a dose-dependent manner. No systematic effect of vitamin C on the CP-B cell line can be detected under acidic conditions. Again, it appears that high levels of vitamin C ameliorate the competitive advantage of the EPC2 cells compared with the BE cells as lower levels of vitamin C do show a substantial effect on competitions between EPC2 and CP-B cells (Figure 3B). Generally, the addition of acid alone favors the BE cells, but the combination of acid and vitamin C results in conditions less favorable for BE cells and more favorable for squamous cells.

### Growth of cell lines in monoculture

**Figure 4:**
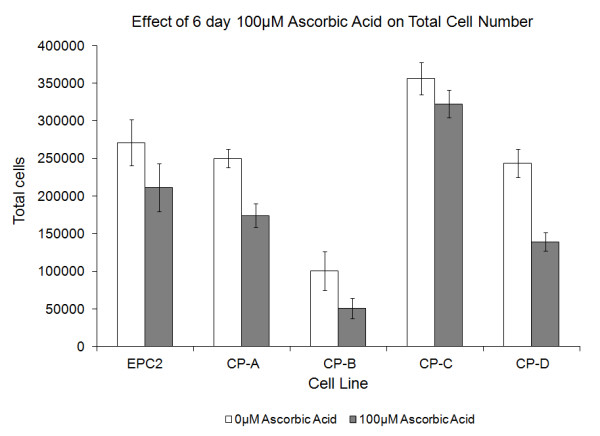
**Effect of Vitamin C Exposure on Cell Number**. The squamous and Barrett's cell lines all show a reduction in average cell number with Vitamin C exposure compared to control conditions. Mean of n ≥ 6 ± standard error of the mean is shown.

Monoculture experiments (Figure 4) demonstrate that vitamin C reduces the net growth for both the squamous and Barrett's cell lines. It is important to note that the monoculture results alone do not explain the results of the competition. In monoculture, for example, the CP-C cell line grows as well or better than the EPC2 squamous line, but in competition the squamous cells quickly outcompete the CP-C cells. It is clear from examination of the monoculture data that the cell lines described here are not acting independently when grown in competition, therefore monoculture data does not accurately describe the co-culture environment. Cell culture competition models are able to capture interactions between different cell types in a way that a monoculture model cannot. This provides direct evidence that competition dynamics are present in the co-culture environment.

### Competitions under varying levels of Vitamin E and EGF

Competitions were also performed with varying levels of Vitamin E and EGF (Additional File 1, Figures S2-S3). EGF did not have a significant effect on the outcome of competition. In the case of vitamin E, very high levels of α-tocopherol strongly suppressed growth of both cell lines. Unexpectedly, the fitness of the squamous cells was more strongly suppressed by vitamin E than the BE cells with the end result that BE cells out-compete squamous cells at high concentrations of Vitamin E (Additional File 1, Figure S2). Lower levels of Vitamin E did not affect the outcome of competition.

### Apoptosis and Proliferation Assays on Vitamin C Competitions

Cell culture competitions provide a window into the net growth of the cells under the conditions defined by the investigator, but they do not identify the mechanism of the fitness effect. While it is clear that vitamin C increases the overall proportion of squamous cells relative to Barrett's cells, it is not clear if this is due to an increase in proliferation/survival of the squamous cells or a reduction in proliferation/survival in BE cell lines.

To determine if the effects of vitamin C in the competition between EPC2 squamous cells and CP-C cells was due to changes in proliferation, differences in levels of phosphorylated serine 10 on histone H3 (PHH3) were measured by flow cytometry. Phosphorylated serine 10 on histone H3 is a marker of mitotic cells. Although the proportion of PHH3+ cells was extremely low, we were able to show reduced proliferation of the CP-C cells under 500 μM ascorbic acid conditions for two separate experiments (Table 1). We did not find a systematic change in proliferation of EPC2 cells with ascorbic acid (data not shown). Several apoptosis assays (cleaved caspase-3, annexin-V+7AAD, TUNEL) were also performed; however, little apoptosis was detected in this system. Because the media which overlays the cells in the competitions is changed every 2-3 days, many apoptotic cells are likely lost as the competition progresses. The caspase-3 assays do hint at an increased apoptosis level for both EPC2 and CP-C cell lines but the total amount of apoptotic cells is very low (< 0.1%, Additional File 1, Figure S4). To overcome the limitation incurred by extrusion of apoptotic cells into the surrounding media, time lapse imaging of cells in competition in the presence of ethidium homodimer-1 was performed. No systematic differences in cell death in the presence of 500 μM vitamin C were detected, nor were there differences in total cell death between monoculture and competition (Additional File 1, Figure S5).

**Table 1:** CP-C Unlabeled BE cells vs. EPC2 DsRed-labeled Squamous Cells

Experiment	Concentration Vitamin C	**Average CP-C % PHH3**^**+ **^**(n = 2)**
1	0 μM	2.64
	500 μM	2.31
2	0 μM	1.32
	500 μM	0.91

We also confirmed that the effect was not due simply to serial passaging of the cells. We were initially concerned that if cell lines adhere to cell culture dishes at varying rates during reseeding, there might be differences in the lag time before exponential growth could resume that might account for differences seen between cell lines in competition. This was of particular concern with vitamin C, which can affect extracellular matrix components [45]. We found no significant differences in "lag time", the time between trypsinization and resumption of exponential growth, between the different cell lines following trypsinization and replating (data not shown).

## Discussion

We have developed a sensitive and robust *in vitro *model of the process of cellular competition, which is thought to drive the evolution of malignancy [1]. The utility of our assay is demonstrated in proof of principle experiments with ascorbic acid, where we find that vitamin C increases the fitness of esophageal squamous cells relative to Barrett's esophagus cells.

The methodology for cell competition is based on bacterial batch culture competition models [46], in which cells are maintained in logarithmic growth and serially passaged. Human cell culture is more complicated than a simple bacterial batch culture system due to the substantially greater potential for interactions between cell lines via mechanisms such as diffusible growth factors, ECM interactions and contact inhibition, although these phenomena are certainly possible in bacterial systems as well [47,48].

The cell culture competition model allows us to capture the relative effect of a compound on 2 cell lines of interest simultaneously. It is this effect, rather than the absolute growth rate, that is most important physiologically as different cell types likely interact with one another. This phenomenon is of particular relevance in Barrett's esophagus, where there is a clear junction between Barrett's cells and normal squamous esophageal cells. It is important to note that in the experiments described here, the normal squamous esophageal cells outcompete the BE cells under almost all concentrations of vitamin C. Therefore, we are comparing the relative rate at which the squamous cells outcompete the BE cells under different concentrations of vitamin C. This baseline advantage of the squamous lines in this system is likely an artifact of the cell culture conditions used. Here, all cells are grown in the serum-free media most suitable for the growth of the normal esophageal squamous cells. The addition of serum in the media leads to increased relative fitness of BE cells compared to no-serum controls in all cell lines tested. In 2 out of 3 cell lines, this increase is such that the BE cells outcompete the squamous cells as would be expected under *in vivo *conditions (Additional File 1, Figure S6). Future models of competition might be developed with a more realistic microenvironment using three-dimensional organotypic cultures that include both extracellular matrix and stromal cells [49-51].

The mechanism of the cancer preventive effect of vitamin C is thought to be via a reduction of reactive oxygen species (ROS). BE is associated with chronic inflammation, which can generate ROS, thought to contribute to the development of a variety of cancers. A meta-analysis and other recent studies have reported that intake of antioxidants such as vitamin C, E, and beta-carotene are inversely associated with risk of esophageal adenocarcinoma [39,40]. While it is clear that there is some relationship between the concentration of antioxidants and BE [e.g. [43]], substantial further experimentation would be required to fully characterize vitamin C as a preventive agent. In general, patients with EA have been found to have lower intakes of antioxidants compared to control populations [52]. Recent studies have shown that BE patients have significantly lower plasma levels of vitamin C (ascorbic acid), xanthophylls and other antioxidants [43]. In addition to lower plasma levels, BE mucosa was found to contain significantly lower levels of vitamin C compared with matched normal squamous mucosa [44]. This reduced presence of exogenous antioxidants may enhance the effects of DNA-damaging oxygen radicals.

Reduction of vitamin C in patients with BE may come from both a diet low in antioxidants as well as a reduced absorption of vitamin C from the stomach [39]. Vitamin C is unstable at non-acidic pH and patients on proton pump inhibitors, such as omeprazole, have more basic gastric fluid. In one study [53], individuals given 40 mg/day for 28 days of omeprazole had a 12.3% decrease in plasma vitamin C levels independent of dietary vitamin C intake, suggesting that there may be lowered bioavailability of vitamin C in patients currently prescribed proton pump inhibitors (PPI). It warrants further investigation whether the current standard of care for individuals with BE, PPI medication (with regular endoscopic surveillance), is promoting reduced levels of vitamin C and whether this may, in turn, promote expansion of the Barrett's segment.

We view these cell culture competition models as particularly useful for early screens of chemoprevention agents, particularly when there are few model systems available, as in the case of BE. Many drugs have subtle effects and this system allows for longitudinal measurement of the effects of various compounds on cell competition. Our model is able to integrate differential fitness effects on both normal and neoplastic cells, regardless of whether they act through decreasing the fitness of neoplastic cells or increasing the fitness of normal cells, and so may be useful for the discovery of new classes of drugs, such as benign cell boosters [2]. In order to screen any large number of therapeutic agents, our model would have to be downscaled to run in 96- or 384-well plates. This is a simple assay designed for an initial screen, performed on plastic in 2D culture, but it can provide preliminary data for more complex animal model testing and for exploration of epidemiologic results.

## Conclusions

We established a cell culture model that can capture the dynamics of competition between two interacting cell lines. Because it is this interaction between normal and neoplastic cells that is the basis for our cancer prevention and therapy interventions, this model can provide an initial screen of potential compounds. We show that ascorbic acid is a modulator of competition between esophageal squamous cells and Barrett's esophagus cells. The advantage of squamous cells relative to Barrett's cells is enhanced by the addition of acid pulses mimicking the reflux conditions of the distal esophagus.

## Competing interests

The authors declare that they have no competing interests.

## Authors' contributions

CM conceived of idea. CM, LM and RK planned all experiments. Competition experiments were performed by RK, LM, and KG. RK and KG carried out proliferation and apoptosis assays. LM performed time lapse imaging. LM performed all statistical analyses and wrote the manuscript. All authors read and approved the final manuscript.

## Pre-publication history

The pre-publication history for this paper can be accessed here:

http://www.biomedcentral.com/1471-2407/11/461/prepub

## Supplementary Material

Additional file 1 Supplemental information Sections 1-6 Click here for file
