# Supplementary material for: An in vitro co-culture model of esophageal cells identifies ascorbic acid as a modulator of cell competition
Source: BMC Cancer. 2011 Oct 25;11:461. doi: 10.1186/1471-2407-11-461 (PMC3213018; doi:10.1186/1471-2407-11-461)
Supplement: Additional file 1 — Supplemental information Sections 1-6 [file 1471-2407-11-461-S1.DOCX]

**Supplemental Information:**

***S1. Test of Neutrality of labeled lines***

We performed a competition between labeled and unlabeled normal squamous (EPC2) cell populations to determine if there is a relative change in label proportion over time and whether or not labeled cell populations respond differently to vitamin C. The GFP labeled cells remain constant relative to the unlabeled cells over 14 days and do not respond to Vitamin C. Mean (n=3) ± standard error of the mean is shown.


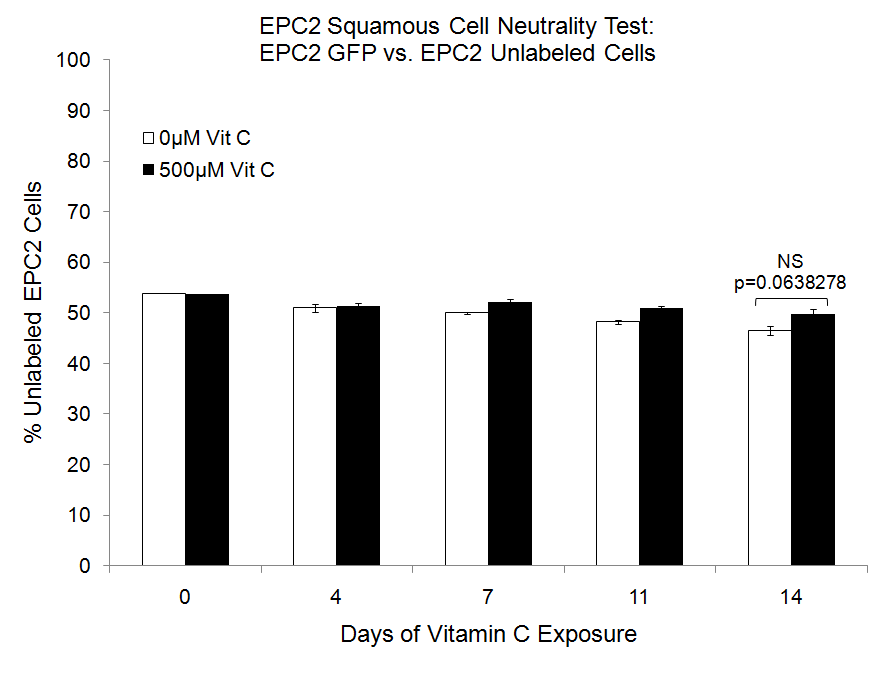


***S2. Test of Effect of Vitamin E***

There are 8 related compounds in the vitamin E family [1], one of which, α-tocopherol, was used for these experiments. While the BE cells appear to do substantially better than the normal squamous cells at high doses, in fact both cells lines were strongly negatively affected by α-tocopherol at this level, with a substantial reduction in cell number apparent at this concentration (data not shown). In these experiments, both the BE and EPC2 cell lines are fluorescently labeled. It is also important to note that vitamin E is a lipid soluble vitamin [2] so may not be amenable to examination in aqueous cell culture media. Mean (n=3) ± standard error of the mean is shown.


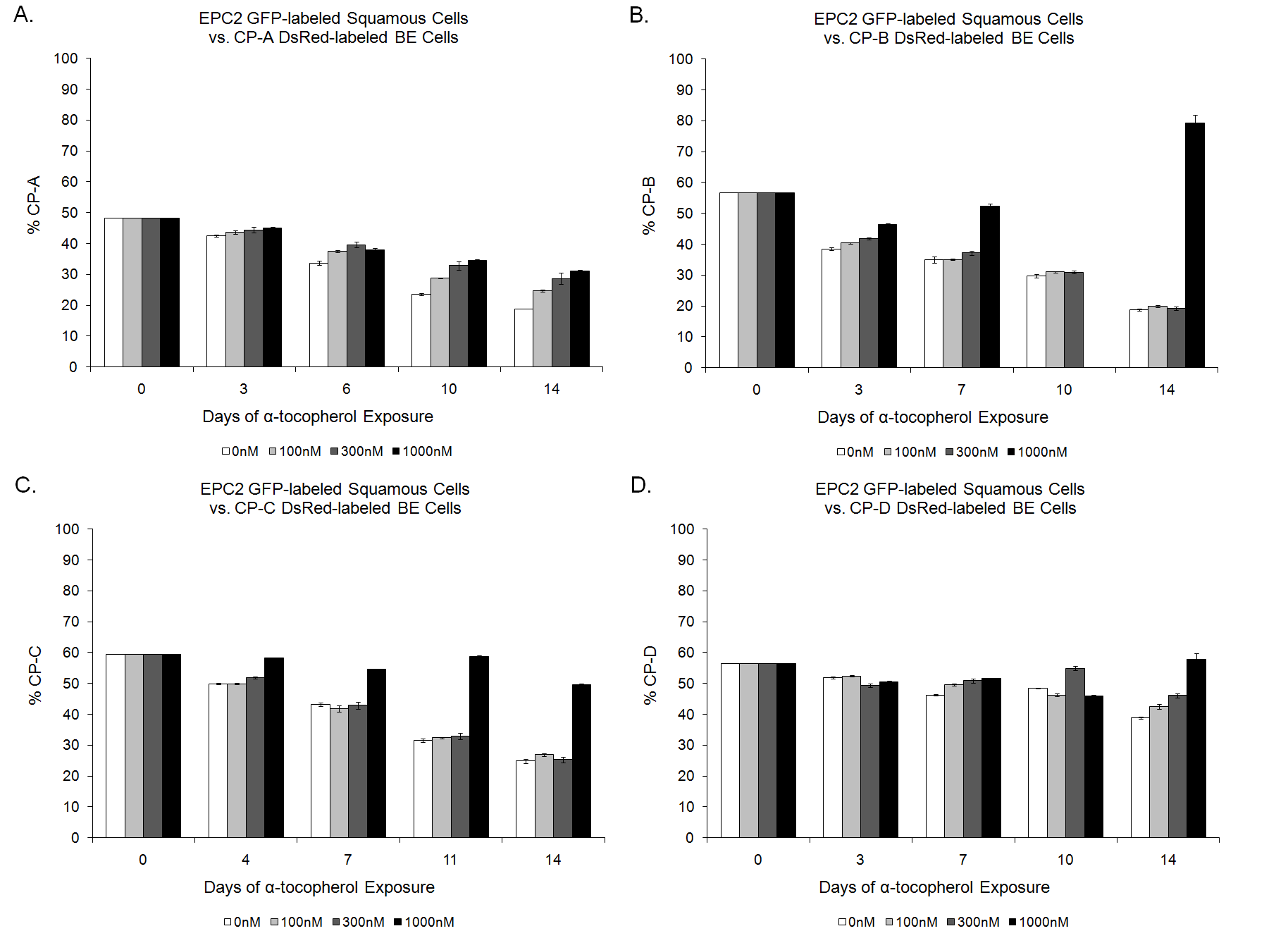


***S3. Test of Effect of EGF***

Here we measured the effect of EGF exposure differing from the standard level of 5ng/mL EGF (Gibco) used in other competitions. Many cancers are fed by growth factors and BE cells are known to have mutations in EGF receptors [3], thus the effect of EGF exposure on cell competition was measured. Over the 14 days of the experiment, no consistent effect of EGF was seen. Mean (n=3) ± standard error of the mean is shown.


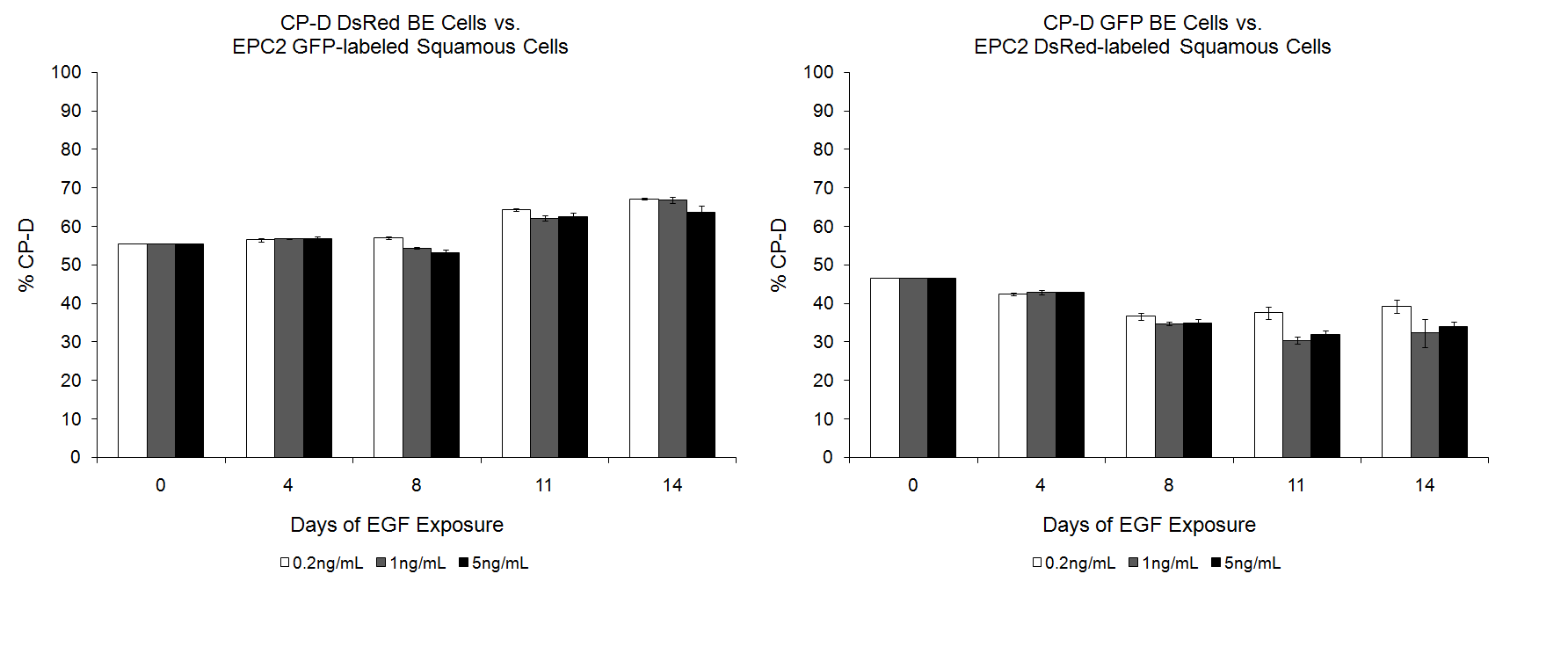


***S4. Cleaved Caspase-3 Apoptosis Assay Results***

BE cell lines did not show a caspase-3 response to the apoptosis inducing compound etoposide (data not shown) and thus were not analyzed for this marker. Etoposide controls demostrate that apoptosis can be successfully detected in EPC2 squamous cells. These cells show an increase in apoptosis with increasing concentrations of ascorbic acid.

| **Etoposide Controls** | |
| --- | --- |
| Etoposide Concentration | Average % Caspase-3^+^ (n=2) |
| EPC2 no antibody control | 0 |
| EPC2 secondary antibody only control | 0.039 |
| EPC2 0uM etoposide | 0.28 |
| EPC2 5uM etoposide | 3.075 |
| EPC2 10uM etoposide | 6.05 |
| EPC2 25uM etoposide | 6.82 |
| EPC2 50uM etoposide | 5.52 |

| Competition | Concentration Vitamin C | Average EPC2 %  Caspase-3^+^ (n=3±S.E.M.) | | |
| --- | --- | --- | --- | --- |
| CP-C vs. EPC2 DsRed | 0µM | 0.036 | ± | 0.007371 |
| CP-C vs. EPC2 DsRed | 50µM | 0.029667 | ± | 0.008667 |
| CP-C vs. EPC2 DsRed | 150µM | 0.052667 | ± | 0.009062 |
| CP-C vs. EPC2 DsRed | 500µM | 0.072333 | ± | 0.012252 |
| CP-D vs. EPC2 DsRed | 0µM | 0.007177 | ± | 0.001912 |
| CP-D vs. EPC2 DsRed | 50µM | 0.017333 | ± | 0.004667 |
| CP-D vs. EPC2 DsRed | 150µM | 0.014667 | ± | 0.00318 |
| CP-D vs. EPC2 DsRed | 500µM | 0.031333 | ± | 0.004372 |

***S5. Time Lapse Imaging of Cell Death***

Extensive time lapse imaging of EPC2 vs. BE cell lines did not show systematic differences in cell death between the cell lines, or between the 0 and 500µM Vitamin C treatments. Too few CP-A cells could be reliably traced in time-lapse images of competition to calculate a reliable % cell death for those, and so instead we calculated the total cell death frequency as well as the EPC2 specific cell death frequency. Percent cell deaths are shown with standard error bars. Total death rate in competition lies between the death rates from the monoculture experiments, and is lower than the EPC2 competition death rates, showing, if anything, CP-As should be gaining a competitive advantage from vitamin C exposure. Thus, differences in cell death rate alone do not explain the outcome of EPC2 cells gaining a competitive advantage from vitamin C exposure.


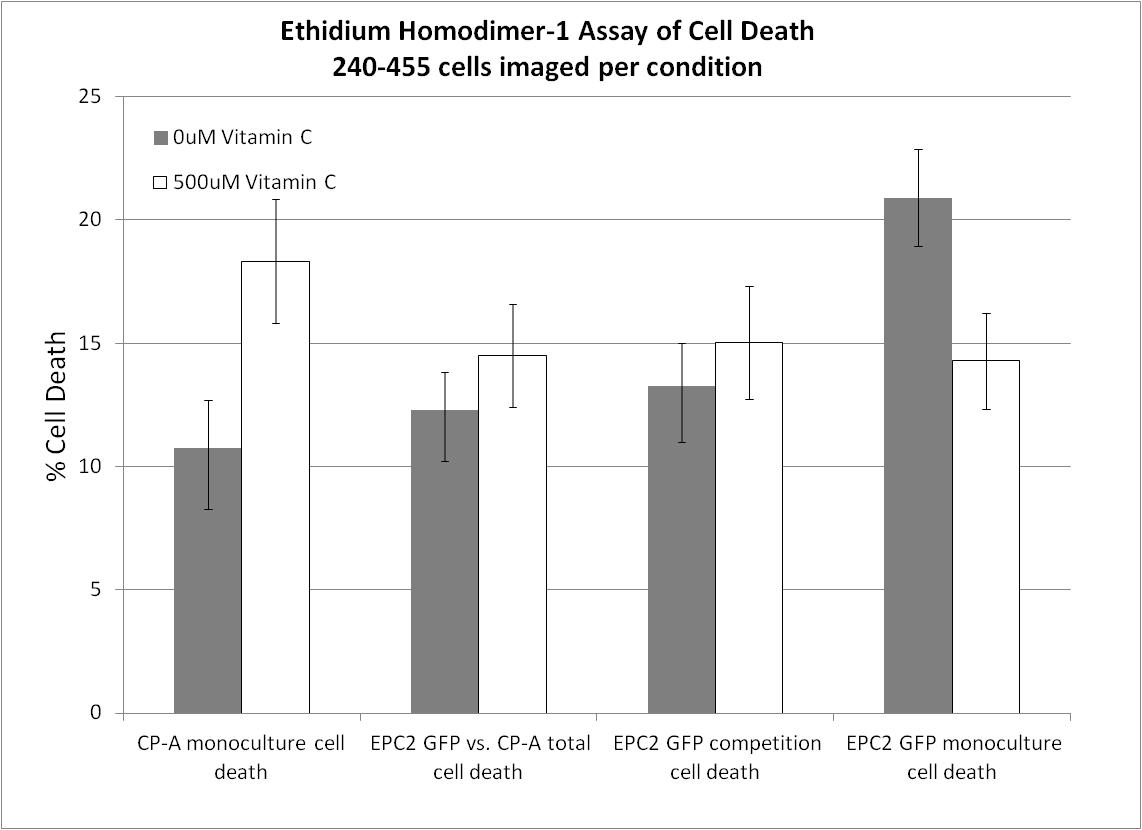


***S6. Test of Effect of Serum***

In all our competitions except CP-D, the EPC2 squamous cells outcompete the BE cell lines using our standard competition methods. This is likely due to the fact that the media used for competition is serum-free and best suited for squamous cells, essentially biasing the outcome of competition in favor of EPC2 cells. Competitions without Vitamin C and with varying amounts of FBS demonstrate that BE cells perform better in an environment with serum. Since different cell lines appeared to respond differently to different amounts of serum, no serum was used in the final vitamin C experiments. In addition, EPC2 squamous cells may differentiate in serum conditions, causing potential bias in the experimental outcome of competition. In these experiments, both the EPC2 and BE cell lines are labeled. Mean (n=3) ± standard error of the mean is shown. This suggests that suppressing the components of serum that benefit BE cells may also be a potential chemoprevention strategy.

***
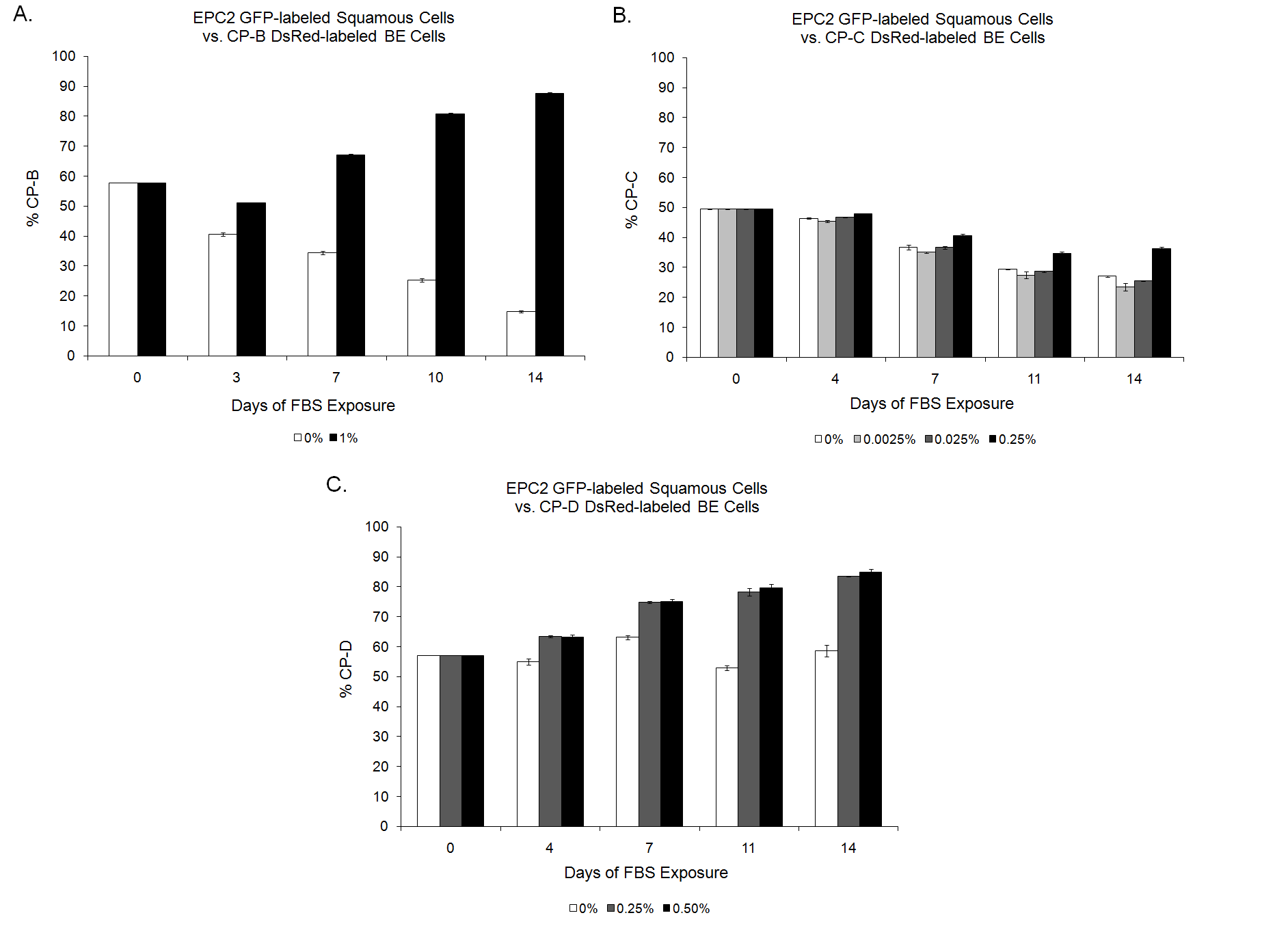
***

***Supplemental References***

1. Ju J, Picinich SC, Yang Z, Zhao Y, Suh N, Kong A-N, Yang CS: **Cancer-preventive activities of tocopherols and tocotrienols**. *Carcinogenesis* 2010, **31**:533-542.

2. Rao CV, Vijayakumar M: **Effect of quercetin, flavonoids and alpha-tocopherol, an antioxidant vitamin, on experimental reflux oesophagitis in rats**. *Eur J Pharmacol* 2008, **589**:233-238.

3. Rygiel AM, Milano F, Ten Kate FJ, Schaap A, Wang KK, Peppelenbosch MP, Bergman JJGHM, Krishnadath KK: **Gains and amplifications of c-myc, EGFR, and 20.q13 loci in the no dysplasia-dysplasia-adenocarcinoma sequence of Barrett’s esophagus**. *Cancer Epidemiol Biomarkers Prev* 2008, **17**:1380-1385.
